# Book Reviews

**Published:** 1807-10

**Authors:** 


### CRITICAL ANALYSIS
### OF THE
### RECENT PUBLICATIONS
### ON THE
### DIFFERENT BRANCHES OF PHYSIC, SURGERY, AND MEDICAL PHILOSOPHY.

---

*Observations on the Application of Lunar Caustic to Strictures in the Urethra and Œsophagus.* By M. W. ANDREWS, M. D. Member of the Royal College of Surgeons of London, late Army Surgeon, and now Physician at Madeira. 8vo. Callow, London, 1807.

EVERY Practitioner, and unfortunately very many men, after arriving at advanced age, are sensible of the frequent occurrences of those irksome diseases which attend the necessary actions of life. We cannot therefore but consider the attention which has for some years past been paid to strictures, as among the many advantages which the present age derives from the improvement of medicine. We shall not enter into the question concerning the danger of applying caustics to a membrane so sensible, and a part so situated as the urethra. Few men ask for assistance in these delicate cases, before they find their situation is become too painful to endure, and still fewer submit to the application of the caustic, till they find the common bougies insufficient to relieve them. If among these dreadful cases, some instances may be stated, in which the caustic may have made a new passage, instead of opening the proper channel to the bladder, it is by no means certain that the patient has been in a worse situation than before: and if we contrast with these, the numerous instances in which, after a long and unsuccessful use of the common bougie, the caustic has restored a miserable sufferer to ease and comfort, there can be no question on which side the balance would preponderate.

Whilst we offer this eulogy in favour of a valuable practice, we are ready to believe that it has been used more indiscriminately than is necessary. That, in many instances, the patient might have found all the benefit from the common bougie that the caustic has produced, and that the rage for a new remedy has induced many unfounded suspicions of strictures, where none existed. This accusation is not meant to be confined to this practice. We have seen the town, and every minor practitioner, equally enraged with purgatives, with calomel, as Sangrado was with bleeding, and, as he describes, the practitioners after him, were with *Kermes*. What does all this prove, but that the *vis medicatrix naturæ*, which some are so much disposed to undervalue, is not so easily opposed as we are apt to believe; and as the candid Syden-

 ham ham acknowledged of the Small-pox, that in a mild case the Doctor cannot kill any more than cure a severe one.

We hope not to be suspected of undervaluing the noble art of Medicine by these remarks. Our only wish is, that practitioners would be careful on all occasions to distinguish those cases in which they claim the merit of a cure, or accuse themselves of having done mischief without any title to either, from such as really have been influenced by their interference. In chronic cases this is neither difficult nor dangerous. In acute diseases, though a quicker decision may be necessary, yet this need never supersede those accurate discriminations, and particularly registers, of the result of our practice. By the help of these, we may join empiricism with physiology, the only rational means of establishing a well founded Theory.

We have been led to these remarks by a transient view of the floating doctrines which pass before us, and not by any thing we have found in Dr. Andrews's performance, that is chargeable with the errors above hinted at. The work begins with two short chapters on the nature and causes of strictures. In these we could wish the author had adopted the distinction made by Mr. Hunter, a writer to whom he always shows a becoming respect. The distinction we allude to, is between the permanent and the spasmodic stricture. The first arising from a gradual, and sometimes insensible contraction of the part; the second from a spasmodic action, which is only temporary, and which as often as it occurs, may be relieved by the common bougie. This distinction is the more necessary, because it is by no means certain, that the spasmodic stricture can be relieved by a caustic, nor that it may not even be increased by it.—We shall not enter into the subject of caruncles any further than to remark, that in cases of very high inflammation, there is no reason why the urethra as well as other mucous membranes should not throw out coagulable lymph; and the accuracy of Mr. Hunter's observations would confirm this opinion. That Gentleman describes the only two cases he had seen as resembling "granulations, or what would be called polypi in other parts of the body."

These, however, are trifling objections against a truly useful practical work, the particulars of which we shall proceed to consider.

The first chapter is on the nature of stricture, on which we have nothing further to remark; the second, on the causes, contains nothing new, but some good cases, illustrating those causes which were remarked by Mr. Home. The third is on the symptoms, which are divided into local and constitutional, both of which are described with sufficient accuracy.

In the fourth chapter the treatment of stricture commences.—In this the author shows the frequent insufficiency of the common bougie to produce a permanent cure, and the advantage derived from the introduction of the caustic, as proposed by Mr. Hunter.

On

On this occasion, Dr. Andrews gives a proof of the goodness of his heart, in paying a proper respect to the merits as well as candour of the late Mr. Hunter. That Gentleman having been accused of claiming the invention, Dr. A. shows that he not only was aware that others had proposed this active remedy, but gives Wiseman the credit of entertaining the same idea, and the probable cause of his relinquishing it. This chapter concludes with a short historical view of the progress of the practice.

The fifth chapter, *on the effects of Strictures in the Urethra and neighbouring parts*, contains some very valuable cases, with highly useful practical remarks. The sixth, states *the objections which have been made to the practice.* This is a very long chapter, and, perhaps, many of its readers will say, much longer than is necessary. It must, however, be recollected, that though in the metropolis we know how little consequence is attached to most of the names introduced to be confuted by the author; yet, in remoter parts of the world, the long titles of a Rowley, and the frequency with which another name occurs, may give an ideal importance to both. Mr. Whateley, however, stands on different ground from either, and we almost doubt whether Dr. Andrews has sufficiently attended to all the advantages that gentleman proposes by the substitution of kali purum for argentum nitratum. It must be admitted, that if the operation of the latter is more sudden, it has the advantage of forming so complete a solution, that a very small slough need be separated afterwards. The solution also being saponacious, may in some degree facilitate the passage of the bougie after it has parted with the kali attached to its end. We only offer this observation as conjectural, nor should we have done this, had we found that Dr. Andrews had actually tried the kali, so as to estimate the comparative merits of the two. Whilst we make these remarks, acknowledging that we have no practical experience to guide us, we feel it our duty to add, that in our opinion, all Dr. Whateley's other objections are satisfactorily answered in the chapter before us, and we could not help feeling pleased whilst we perused it, at the zeal which our author shows in defending his master.

The following may serve as a specimen of this as well as of the author's style.

" I cannot conclude this chapter, without making one general remark on Mr. Whateley's observations, relative to the treatment recommended and practiced by Mr. Home. Throughout the whole of them, he is continually making Mr. Home " thrust his large armed bougie through the strictures, until he can obtain a free passage to the bladder." I apprehend this has arisen from Mr. Home, in his work, having frequently used the word " through ;" as " the armed bougie passed through the stricture, and the caustic was applied to a second," &c. Mr. H. however, does not mean it to be understood that the force, which he employed, made the bougie pass through the contracted part of the canal, but merely merely that the action of the caustic had rendered that point sufficiently pervious to admit both bougies being passed further on. That this is his meaning is very evident, and I should not have conceived it possible for any one to have misunderstood it.

"In p. 108 of Mr. Whateley's Treatise, we find the following remark: "It appears highly probable, therefore, that the caustic 'bougie, as used by Mr. Home, is, for want of such extension of 'the penis in cases of this kind, prevented from acting directly on 'the stricture; and that, in consequence of its hitching on some 'part of the urethra, it may sometimes be forced into the sub'stance of the corpus spongiosum, instead of making its way 'through the stricture," &c. &c.

"Mr. Home, in his lectures, is very particular in his directions for passing the armed bougie, particularly when applied to a stricture at seven inches; and, in his Treatise, he recommends the frequent passing of a catheter more curved than ordinary, in those cases that require a great number of applications to the stricture at this point, or when there appears to be any irregularity in the urethra.

"Upon the whole, it would appear that Mr. W's remarks are levelled against Mr. Home, without reflecting that the failure and unpleasant occurrences, in many instances, are owing to the mismanagement of cases in the hands of inexperienced practitioners, and frequently to the imprudence of patients themselves.

"This is evidently intended to overturn one mode of practice by introducing another, which differs only in the kind of caustic used, and the manner of applying it, as he says in p. 127. "At 'the same time there is no doubt, but that many sound cures 'have been made by Mr. Home's mode of treatment; but I con'tend that these might have been effected by a method of apply'ing caustic, without the risk of producing that dreadful train of 'effects enumerated by him, &c. &c."

The seventh chapter contains thirty-two cases of stricture, relieved by the Lunar Caustic: in some of these, the unarmed bougie had been ineffectually used.

The eighth and last, is a short chapter, containing three cases of stricture in the œsophagus. The first and second are the most valuable, though not the most successfully treated. But they contain the account of the appearances after death, which cannot be so well understood without the annexed drawings. We shall therefore transcribe the last.

"Case III.—A poor woman, about 50 years of age, applied to me in April, 1805. Since August, 1804, she had experienced great difficulty in swallowing any thing, either fluid or solid, and, within the last ten weeks, could get nothing into the stomach but what was perfectly fluid; any attempt to swallow solids produced vomiting.

"On the 18th of April, I applied the caustic, and repeated it on the 19th and 20th. On the 21st, I passed an unarmed bougie into into the stomach; on the 22d, 23d, and 25th, I passed a probang with great ease, and, as she swallowed tolerably well, I allowed her to return to her friends in the country.

"In May following she came back to me, as she again found great difficulty in swallowing, the act often producing vomiting; on the 11th, I applied the caustic, and repeated it on the 12th and 15th, after which she went into the country perfectly relieved."

Such are the contents of Dr. Andrews's work, as far as our limits will permit us to pourtray it. If it is neither adorned with brilliancy, nor enriched with novelty, it contains many useful practical remarks, is written with becoming modesty, and gives us reason to hope that his sojournment in the Island of Madeira, will be useful to science as well as to the inhabitants of that favoured spot.

---

*Pethox Parvus. Dedicated, without Permission, to the Remnant of Blind Priests of that Idolatory.* By ICONOCLASTIS, 8vo. Murray, 1807.

THE object of this little brochure, is to ridicule those who still retain their attachment to Small-pox Inoculation. But it should be recollected, that ridicule is so far from being the test of truth, that for the most part it is more commonly resorted to when truth is too powerful for those who use such random weapons. We therefore conceive so good a cause is rather injured by such a performance, especially when ushered in without a name. Our opinion of vaccination is too well known to be repeated, but we never wish to encourage that illiberal spirit which would misinterpret the best endeavours of benevolent characters. We transcribe the following note, to show the spirit of the author, and in justification of our own remarks.

"Hospitals were originally founded by humane men to preserve the lives of the poor: but the Small-pox Hospital so far deviates from this purpose, that it has become one of the principal sources of mortality in this town. Above 2000 persons are infected annually with a contagious disease at this house, which is likewise a market where infectious matter is sold at an unconscionable price to all who apply for it; by which means the contagion is perpetually disseminated through all the streets, lanes, and narrow courts of this crowded city. It is difficult to judge of the motives which actuate the Governors. Do they believe it charity to bestow death? And do they agree with Mr. Malthus, in considering it a benevolent act to sweep away a redundant population? Whatever are their motives, we are astonished that any medical men should consent to be the executioners of this species of patriotism. We hope the legislature will soon relieve them from any notion of political duty they may entertain, and will snatch the murderous lancet from their hands."

Now it appears by the Report of the College of Surgeons, that out out of 164,381 who have been vaccinated by their own members, nearly one in a thousand cases have been followed by subsequent small-pox, or eruptions consequent on the introduction of vaccine virus; without reckoning any occurrences of small-pox, excepting in such as have been vaccinated by the surgeon reporting the facts. This is enough to alarm those whose nearer connections have been the subjects of such untoward events, and to them some little delicacy is due. As to the propriety of preventing small-pox inoculation, we shall only remark, that, however proper it may be, it does not appear how it should be more necessary now, than before the introduction of vaccination.

---

*The Ancient and Modern History of Nice, comprehending an Account of the Foundation of Marseilles; to which are prefixed, Descriptive Observations on the Nature, Produce, and Climate of the Territory of the former City, and its adjoining Towns, with an Introduction, containing Hints of Advice to Invalids, who, with the hope of arresting the Progress of Disease, seek the renovating Influence of these salubrious Climes.* By J. B. Davies, M. D. one of the British Captives, at Verdun; Author of the Project de Reglement concernant les Déces, and Member of several Medical Societies, 8vo. London, 1807.

Nothing is more unthankful to a Physician, than the office of recommending change of climate to a patient. If the patient wishes to go, he will interpret the Doctor's opinion accordingly: if not, he will stay so late after the advice is given him, that the best thing he could do, would be to stay at home. The present work shows, with a most desirable accuracy, every part of the question, and describes the climate and accommodations of Nice, as well as the particulars attending the voyage, with a minuteness highly necessary for the class of readers for whom it is intended.

The rest of the work not being connected with the object of our labours, we shall omit to notice.

---

*A Letter containing some Observations on Fractures of the Lower Limbs. To which is added, an Account of a Contrivance to administer Cleanliness and Comfort to the Bed-ridden, or Persons confined to Bed by Age, Accident, Sickness, or any other Infirmity, with explanatory Plates.* By Sir James Earle, F. R. S. Surgeon Extraordinary to his Majesty, and Senior Surgeon to St. Bartholomew's Hospital, 8vo. Callow, London, 1807.

It is so difficult to give an accurate account of any machinery, without plates, that we shall only remark the present attempt to relieve the irksomeness and pain of those who are confined to their beds, does equal credit to the author's head and heart.

*A. Rowland*

*A Rowland for an Oliver, in Answer to Dr. Moseley's Oliver for a Rowland, and to Mr. Birch: containing a Defence of Vaccination.* By John Ring, Member of the Royal College of Surgeons in London, and of the Medical Societies of London and Paris.

It has been remarked, that contests with the pen differ from those with the sword or pistol, not more in the weapons than in the progress and termination. In the latter, the victor remains last in the field; in the former, the vanquished. In the latter the weapons and ammunition are made as similar and equal as possible; in the former, each combatant is at liberty to use poisoned arrows and unfair arms: but instead of their giving any advantage in the contest, it is generally observed that they demonstrate the weakness of the champion, and lead to speedy defeat and disgrace. These weapons wound their Author more by their recoil, than the enemy against whom they are directed. Impressed with these sentiments, we wish Mr. Ring had left his adversaries to perish by their own arms, rather than to have put himself on a level with them, and thereby exalted them into some notice.

